# Camrelizumab Combined With Gemcitabine and Albumin-Bound Paclitaxel for Neoadjuvant Therapy in the Treatment of Progressive Gallbladder Cancer: A Case Report

**DOI:** 10.3389/fonc.2022.818626

**Published:** 2022-03-14

**Authors:** Jing Wu, Zheng Wang, Jing Li, Xue-Hui Peng, Yi-Chen Tang, Xiao-Bing Huang, Yong-Gang He

**Affiliations:** Department of Hepatobiliary Surgery, The Second Affiliated Hospital of Army Medical University, Chongqing, China

**Keywords:** neoadjuvant therapy, immune checkpoint inhibitor, gallbladder cancer, chemotherapy, AG

## Abstract

**Background:**

The roles of immune checkpoint inhibitors in the treatment of gallbladder cancer are still unclear and challenged by controversial findings. Recent research has shown that immune checkpoint inhibitors in combination with chemotherapy may alleviate disease progression.

**Case Summary:**

A 45-year-old female patient with gallbladder cancer accompanied by multiple abdominal lymph node metastasis was treated with camrelizumab combined with paclitaxel for injection (albumin-bound) and gemcitabine (AG) to downstage the tumor before a radical surgery could be performed. The postoperative quality of life was superior to the preoperative level.

**Conclusion:**

Camrelizumab + AG offers a new therapeutic option for gallbladder cancer with multiple abdominal lymph node metastasis, which, however, warrants further validation in clinical trials.

## Introduction

Gallbladder cancer is a common malignancy in the biliary system, and surgery offers the best chance for a cure (1). However, gallbladder cancer (GBC) is featured by difficulties in early diagnosis, rapid tumor progression, high degree of malignancy, easy recurrence/metastasis, and poor prognosis. Most patients would have already missed the opportunity for surgical resection at the time of diagnosis (1). Neoadjuvant therapy has provided a new treatment option for patients with unresectable advanced malignant tumors, which may downstage the tumor and prolong the survival time of patients. Neoadjuvant therapy may also increase the possibility of achieving a successful complete resection (2). At present, chemotherapy combined with gemcitabine + cisplatin (GC) is the standard treatment strategy for patients with advanced GBC (3). Research has shown that GC combined with paclitaxel for injection (albumin-bound) (PAB) prolonged the progression-free survival (PFS) and overall survival (OS) (4). PAB + gemcitabine (AG regimen) has been applied for the treatment of advanced biliary cancer (5). Notably, all mismatch repair protein (microsatellite instability, MSI)/deficient mismatch repair protein (dMMR)-positive tumors can be treated with immune checkpoint inhibitors (6).

Preclinical studies have found that chemotherapeutic drugs could enhance the endogenous immune response through multiple mechanisms: firstly, chemotherapeutic drugs may activate the adaptive immune system by increasing the expression of human leukocyte antigen (HLA) and enhancing the stimulation of T cells; secondly, chemotherapeutic drugs may recover the immunological surveillance by disrupting STAT6-mediated immunosuppression (1). Based on these theories, a combination of immunotherapy and chemotherapy has been offered for patients with advanced biliary tract cancer (BTC) in the recent years and has shown promising efficacy (1, 7).

According to previous studies, neoadjuvant chemotherapy for degrading before surgical treatment will benefit advanced carcinoma patients. Here, we report on a case of GBC with multiple local lymph node metastasis who has been successfully treated with camrelizumab + AG.

## Case Presentation

**Figure 1:**
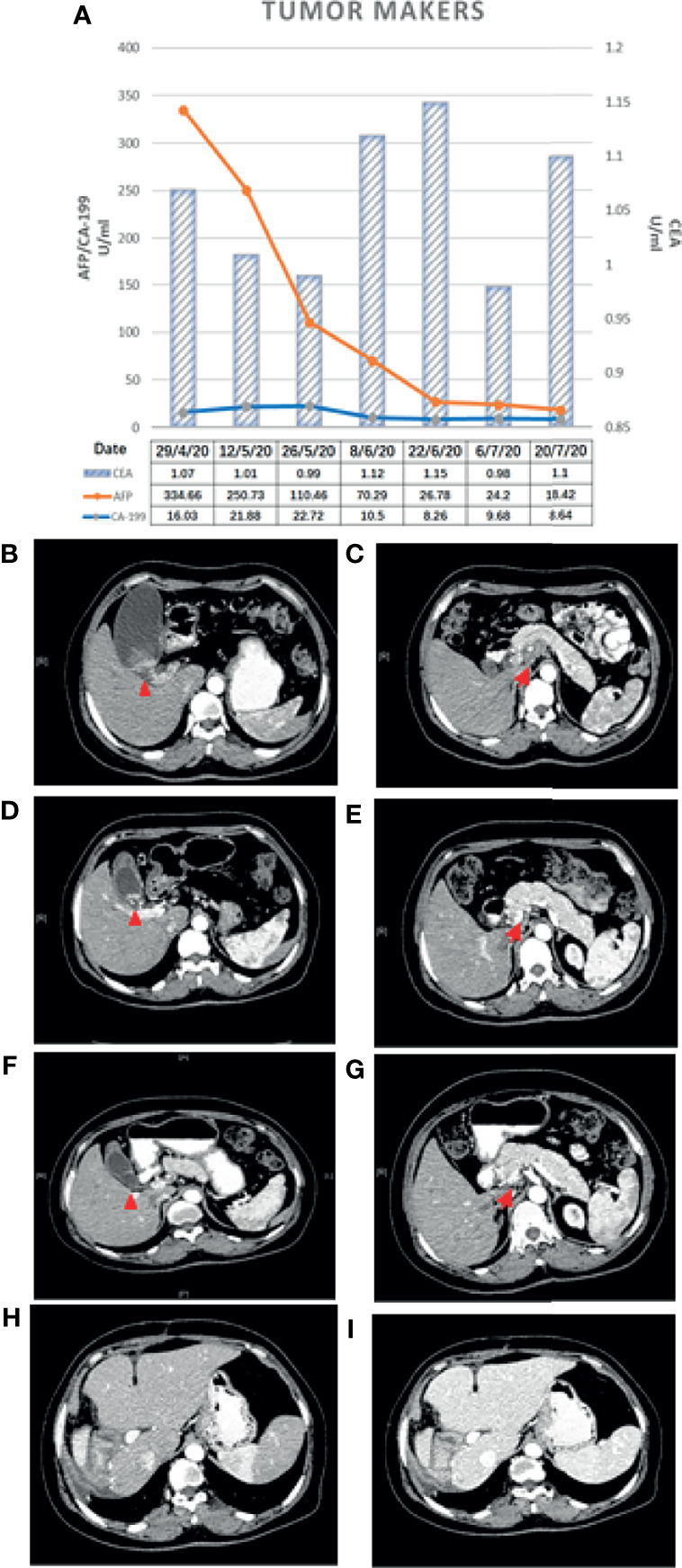
**(A)** Changes in the tumor makers. **(B**, **C)** Baseline CT scan. **(D**, **E)** CT scan after the first treatment cycle. **(F**, **G)** CT scan after the second treatment cycle. Changes in CT findings: the primary gallbladder cancer and metastatic lymph nodes shrank after treatment with gemcitabine + albumin-bound paclitaxel combined with camrelizumab for two cycles. **(H**, **I)** CT examination after surgery. No recurrence or metastasis was found on abdominal CT at 14 months. The *red triangle* denotes the primary lesion and the *red arrow* indicates the metastatic lymph nodes posterior to the pancreas.

A 45-year-old woman was hospitalized because of right upper abdominal pain for 3 months. The patient had a cesarean delivery 20 years ago. The patient had no prior history of cancer and chronic viral hepatitis and no family history of BTC or other hereditary diseases related to the gallbladder or liver. Tenderness was examined in the right upper abdomen. The level of alpha-fetoprotein (AFP) was 334.66 ng/ml. The levels of carcinoembryonic antigen (CEA) and carbohydrate antigen 19-9 (CA 19-9) were within normal ranges (**Figure 1A**).

**Figure 2:**
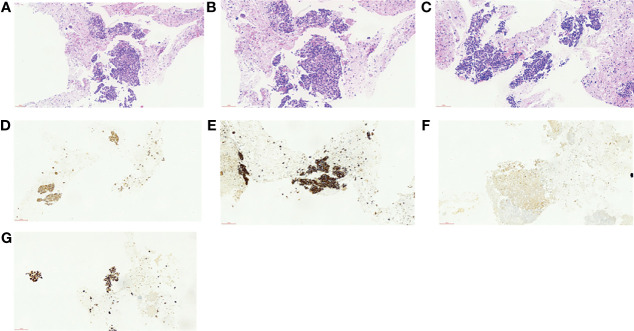
**(A**–**F)** Results of histopathology before neoadjuvant therapy. **(A)** Hematoxylin–eosin (HE), ×40. **(B)** HE, ×200. **(C)** HE, ×200. **(D)** Cytokeratin 18 (CK18), ×200. **(E)** Pan cytokeratin (CK-pan), ×200. **(F, G)** Fibroblast activation protein (FAP), ×200.

Abdominal contrast-enhanced computed tomography (CT) and magnetic resonance imaging (MRI) revealed GBC (3.2 cm × 2.7 cm in size) accompanied by localized multiple lymph node metastasis (the largest one was 2.5 cm × 3.6 cm) posterior to the pancreas, gallbladder enlargement, and gallbladder stones (**Figures 1B, C**). PET-CT indicated a cervical nodule shadow (approximately 3 cm in diameter) and increased FDG metabolism. All the aforementioned combined with the medical history indicated a primary tumor. In addition, a nodule shadow in the rear of the pancreatic head (approximately 2.5 × 3 cm in diameter), multiple primary lymph nodes in the para-abdominal aorta, and increased FDG metabolism indicated metastatic disease in these organs. PET/CT did not detect metastasis in other distant organs. Endoscopic ultrasound-guided fine-needle aspiration revealed positive lymph nodes posterior to the pancreas and a space-occupying lesion in the gallbladder. Ultrasonic endoscopy found one approximately 2.5 × 3-cm hypoechoic envelope block (without internal blood flow signal) and another approximately 3-cm moderate echo envelope block near the neck (without internal blood flow signal). The local gallbladder wall was not smooth, with a 2.3-cm stone at the bottom of the gallbladder. The puncture biopsy tissue was reddish brown. The presence of heterogeneous epithelioid cell groups combined with the immunohistochemistry (IHC) results supported the diagnosis of a poorly differentiated adenocarcinoma, although the possibility of neuroendocrine differentiation cannot be completely ruled out. The IHC results showed the following: CK+, Ki-67 (70%–80%)+, CK18+, Villin+, CgA a small amount+, SYN−, CD56−, TTF-1−, Hepatocyte−, AFP−, CDX-2−, CD34−, CA 19-9− (**Figures 2A–G**).

### Final Diagnosis

No other obvious signs were shown. The examinations and imaging findings all indicated signs of GBC. According to the American Joint Committee on Cancer (AJCC) guidelines (8th edition), it was evaluated as T3N2M0 (IIIA) stage, for which surgical resection was not recommended.

### Treatment

A multidisciplinary team (MDT) recommended the application of multidisciplinary treatment, for which genetic tests would be helpful. However, the patient refused to undergo genetic testing due to financial difficulties and requested to be treated directly with chemotherapy plus immunotherapy as soon as possible.

**Figure 3:**
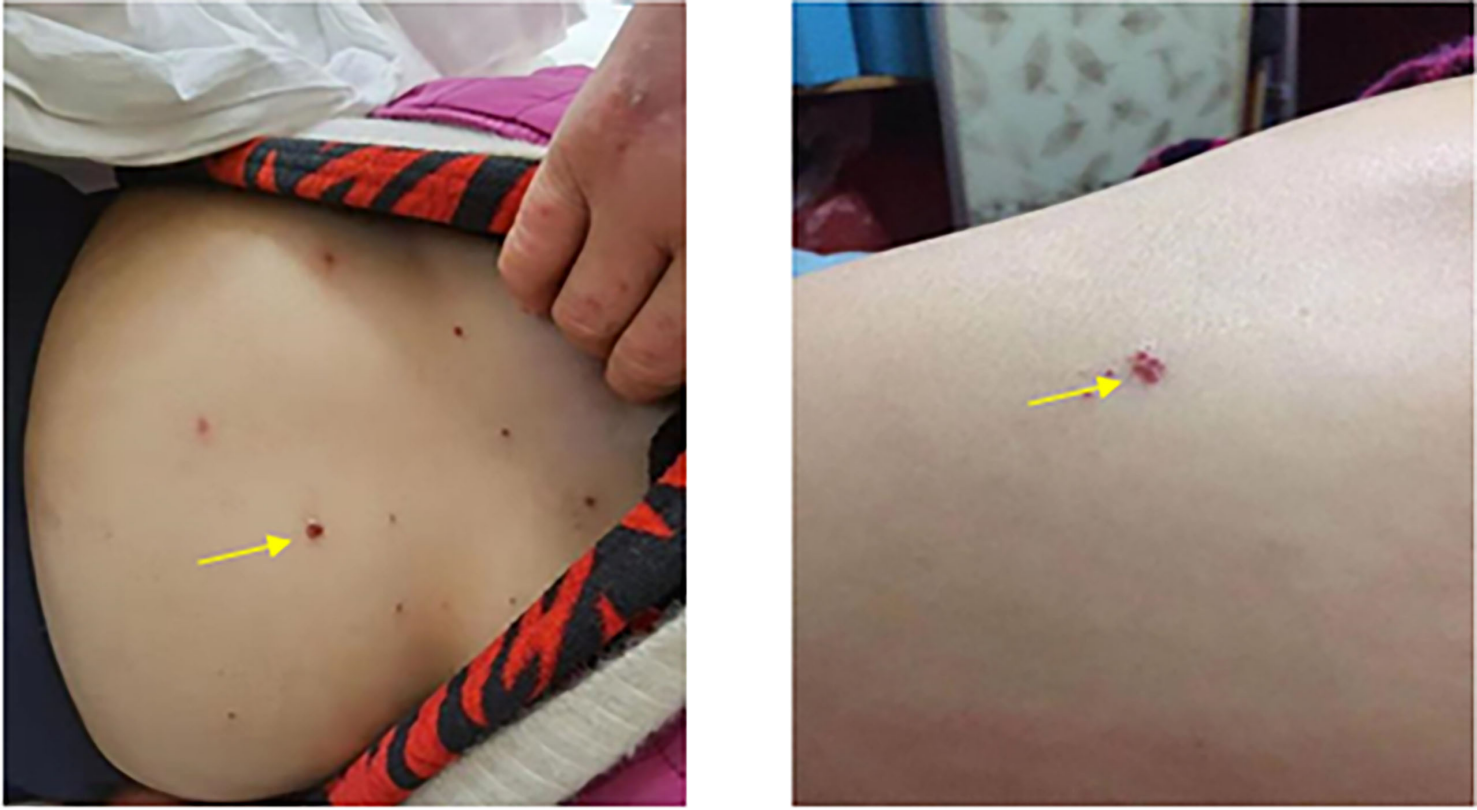
Reactive cutaneous capillary endothelial proliferation (RCCEP) (*yellow arrows*) following immunotherapy.

According to the guideline of the Chinese Society of Clinical Oncology (CSCO), the results of a randomized controlled phase III clinical trial did not support the benefit of neoadjuvant chemotherapy for biliary tract malignant tumor. Patients were recommended to attend the clinical trial. Referring to the literature reports and the CSCO guideline (5), the following treatment protocol was then chosen: 1,000 mg/m^2^ gemcitabine (Stockhausen Pharmaceutical Co., Ltd., Jiangsu, China) plus 125 mg/m^2^ PAB (Shiyao Group Ouyi Pharmaceutical Co., Ltd., Hebei, China) combined with 200 mg camrelizumab (an immune checkpoint inhibitor) (Hengrui Medicine Co., Ltd., Jiangsu, China). The patient was treated with 1,000 mg gemcitabine and 170 mg PAB on days 1, 8, and 15 and with 200 mg camrelizumab on day 1 every 3 weeks. The next cycle of treatment was repeated after a 2-week interval. Abdominal CT and measurements of the tumor markers were performed after each treatment cycle to assess the efficacy (**Figures 1D–G**) using the Response Evaluation Criteria in Solid Tumors (RECIST), version 1.1. After two cycles of treatment, the gallbladder tumor and metastatic lymph nodes shrank more than 30% (partial response, PR), and the AFP level decreased from 334.66 to 24.2 ng/ml. The tumor was then evaluated as resectable. During the therapy, the patient had good appetite without nausea and vomiting and scored 0 in the Eastern Cooperative Oncology Group (ECOG). Drug-induced myelosuppression was noted, as the white blood cell (WBC) count decreased to 2.14 × 10^9^/L. The patient also experienced other hematologic toxicities during the treatment (**Table 1**). These adverse effects improved after subcutaneous injection of recombinant human granulocyte–macrophage colony-stimulating factors. In addition, the patient suffered from generalized pruritus and hemangioma-like changes at the forearms, back, and knee joints after the first dose in the second cycle (**Figure 3**), which were not alleviated after administration of loratadine (i.e., an anti-allergic drug). However, these symptoms were improved after intravenous infusion of 5 mg dexamethasone. The therapeutic tolerance decreased after the second cycle. After consultations with the MDT, the patient was encouraged to continue the current treatment protocol. However, considering the decreased tolerance to chemotherapy and the option of surgical resection, the patient refused further chemotherapy plus immunotherapy and requested a radical surgery. After preoperative examinations and informed consent of the patient, radical surgery was performed to remove the primary tumor, along with regional lymph node dissection under general anesthesia. The patient was also informed that hemihepatectomy + biliary–intestinal anastomosis or even pancreaticoduodenectomy might be performed during the surgery, depending on specific pathological findings. After informed consent was obtained from the patient’s family, the surgery was performed under general anesthesia on August 10, 2020. During the surgery, the gallbladder was removed, and a cystic duct margin was found positive after a frozen section pathological examination. Therefore, a partial bile duct resection was performed. Intraoperative pathological examination showed that the first resection margin of the common bile duct was positive and the second lower resection margin of the common bile duct was negative. In addition, the resection margin was positive for the right hepatic duct and negative for the left hepatic duct. According to the intraoperative pathological examination results, the surgical procedures were changed to right hemihepatectomy, radical cholecystectomy, and regional lymph node dissection, with informed consent of the family because the patient was unconscious.

**Table 1:** Hematological and non-hematological toxicities during neoadjuvant therapy (grading according to CTCAE 3.0).

Hematological toxicities	Baseline	Grade	Non-hematological toxicities	Baseline	Grade
White blood cells	0	II	Nausea	0	I
Neutrophils (%)	0	I	Vomiting	0	I
Hemoglobin	0	I	Fatigue	0	I
Platelets	0	I	Pruritus	0	II

After the surgery, the patient developed pulmonary infection and pleural effusion due to prolonged immobility, which were alleviated after antimicrobial therapy and ultrasound-guided thoracentesis and drainage. The patient was discharged on September 11, 2020. Postoperative pathology revealed that the lesion was a moderately differentiated adenocarcinoma of the gallbladder (**Figures 2D, E**) affecting the entire wall of the gallbladder, along with visible choroidal carcinoma thrombosis and nerve invasion. Metastases were found in lymph node stations 8 and 9, but not in stations 12, 14, and 16 (7). As it had been reported that postoperative adjuvant therapy could improve OS, the CSCO guideline recommended the use of capecitabine or participation in other clinical trials. Since the neoadjuvant chemotherapy plan of the patient was effective before surgery, we continued to treat this patient with chemotherapy plus immunotherapy after surgery. So far, the PFS of this patient has reached 14 months. The patient has maintained good quality of life in the past 9 months without disease progression (**Figures 1H, I**).

## Discussion

Most GBC patients are diagnosed at an advanced stage. The benefit of radiotherapy in the clinical treatment of GBC still needs further confirmation. As a result, the value of chemotherapy as a treatment option second only to surgery for GBC has been increasingly recognized (8). Gemcitabine + cisplatin has been reported as the first-line chemotherapy for GBC, but has a response rate of only about 20% (8). Most patients will suffer from disease progression soon after chemotherapy. Gallbladder cancer patients typically cannot tolerate high-intensity, long-duration chemotherapy due to various factors such as age, physical status, disease severity, and tumor burden. Therefore, for patients with inoperable GBC, a high-efficacy and low-toxicity chemotherapy regimen is particularly important.

For patients with advanced BTC, adding PAB to GC could achieve longer median progression-free survival (mPFS; 11.8 months) and median overall survival (mOS; 19.2 months) than the conventional GC regimen alone, with the mPFS and mOS in the GBC subgroup being 4.1 and 15.7 months, respectively (4). Treatment with PAB in combination with GC prolonged the mPFS and mOS compared with controls treated with GC alone (4).

For unresectable or metastatic extrahepatic cholangiocarcinoma, gemcitabine combined with PAB is feasible. In a phase II trial, Sahai et al. (4) found that PAB + gemcitabine was well tolerated when used as the first-line treatment for advanced or metastatic cholangiocarcinoma and concluded that this regimen might be an alternative option to the current therapeutic approaches for advanced cholangiocarcinoma.

The immunotherapy for cholangiocarcinoma included cancer vaccines, adoptive cellular immunotherapy, and immune checkpoint inhibitors (9). Some studies suggested that tumor vaccines combined with chemotherapy may increase the response rates of patients with BTC, including GBC, but validation in large-scale studies is lacking (1). Immune checkpoint inhibitors have become a new hotspot in current cancer research. By blocking specific pathways, these agents can release tumor-induced immunosuppression and activate the specific immune response to cancer cells, thus achieving immune-mediated clearance of cancer cells. Such efficacies are based on the effects on immunosuppression and tumor immune escape mediated by the interaction of programmed cell death 1 (PD-1), cytotoxic T-lymphocyte antigen 4 (CTLA-4), and their ligands. Camrelizumab (AiRuiKa™) is an immune checkpoint inhibitor. It binds to the PD-1 receptor, blocks the binding between PD-1 and programmed death-ligand 1 (PD-L1) to wash and activate T cells, and produces sustained antitumor effects to inhibit tumor growth (10–12). Currently, camrelizumab has been used for the treatment of a variety of malignancies (13).

Wang et al. (14) reported that PD-1/PD-L1 blocking therapy may cause immune-related adverse reactions (immune-related adverse events, irAEs) and, hence, proposed combining immunotherapy with antitumor drugs to improve the therapeutic effect. However, there have been concerns that the combination therapy may also increase the incidence of more complex treatment-related adverse events. Chen et al. used camrelizumab + gemcitabine and oxaliplatin (GEMOX) to treat patients with advanced BTC and achieved promising outcomes with tolerable toxicities (8). Patients with GBC seemed to benefit more from this treatment. Gou et al. (15) reported that patients with advanced BTC who received a PD-1 inhibitor combined with a chemotherapy regimen had longer PFS (5.8 months) than those who received chemotherapy alone (3.2 months), providing supportive evidence for the efficacy of a combination treatment regimen for advanced BTC. Sun et al. (1) reported that the median OS of the PD-1 inhibitor combined with chemotherapy (14.9 months) was significantly longer than that of the PD-1 inhibitor monotherapy (4.1 months) and of the chemotherapy monotherapy (6.0 months). The combination of PD-1 inhibitor and chemotherapy also showed significantly longer median PFS (5.1 months) than that of PD-1 inhibitor monotherapy (2.2 months) and chemotherapy monotherapy (2.4 months). However, a considerable number of patients did not benefit from this therapy, which may also be associated with serious adverse events. Therefore, evaluation of predictive biomarkers of individual tumor tissues before therapy initiation is critical. Tumor mutation burden, mismatch repair genes, and MSI may be predictors of immunotherapy (16). Recent studies have found that the peripheral blood levels of circulating hematologic and serum cytokines and MAGE1 might serve as potential cancer biomarkers (17–19). Albrecht et al. (20) reported that there is limited evidence on the efficacy of PD-1 inhibitors combined with other chemotherapeutic agents for GBC. The authors also reported that PD-L1 is upregulated in tumors and immune cells in a subpopulation of advanced Western GBC, providing evidence that the TIGIT/CD155 axis is a novel immune checkpoint for complement therapy for GBC. This further confirms the need for a prospective study of PD-L1 in the treatment of advanced GBC.

Tumor mutation burden, mismatch repair genes, and high MSI may be predictive factors for immunotherapy. In this article, we reported our experience in treating a case of GBC with lymph node metastasis with camrelizumab + AG. Chemotherapy plus immunotherapy may have different tolerability profiles, such as bone marrow suppression and loss of appetite after chemotherapy. In addition to the many irAEs, camrelizumab also has a unique adverse event: reactive cutaneous capillary endothelial proliferation (RCCEP) (12). Our patient suffered from myelosuppression and the WBC count declined after the chemotherapy, which was alleviated after administration of recombinant human granulocyte colony-stimulating factor. In addition, RCCEP occurred in the trunk and extremities (**Figure 3**). After hormone therapy, RCCEP in the limb was slightly relieved. After discontinuation of camrelizumab, RCCEP was resolved and the patient’s quality of life improved. The patient has been followed up for 14 months; currently, there is no tumor recurrence, the levels of CEA, CA19-9, and AFP are normal, and the quality of life is good.

Chemotherapy plus immunotherapy achieved definite efficacy in this patient, but the patient showed poor tolerance. The patient’s physical tolerance decreased significantly after completing the second treatment cycle and requested direct surgery. Before the initiation of neoadjuvant therapy, the pathological diagnosis of the lesion was a poorly differentiated adenocarcinoma. After six sessions of neoadjuvant therapy, the gallbladder tumor and metastatic lymph nodes were reduced in size, and radical surgery was performed. Postoperative pathology revealed that the lesion was a moderately differentiated adenocarcinoma of the gallbladder, suggesting that the tumor had been downstaged after neoadjuvant therapy.

In this case, the patient was initially diagnosed with GBC with localized multiple lymph node metastasis, which made surgery infeasible. However, successful surgical resection was performed after treatment with camrelizumab + AG, and the patient has remained recurrence-free 14 months after surgery.

## Conclusion

Locally advanced GBC could be downgraded after neoadjuvant therapy, which may improve the feasibility of performing a surgery. This strategy provides a new treatment option for GBC patients to prolong survival time and improve their quality of life. However, more accumulation of cases is necessary, and our finding needs to be further validated in large multicenter studies.

## Data Availability Statement

The original contributions presented in the study are included in the article/supplementary material. Further inquiries can be directed to the corresponding authors.

## Ethics Statement

Informed written consent was obtained from the patient for the publication of this report and any accompanying images.

## Author Contributions

JW, ZW, X-BH, Y-GH, and JL contributed to the conception and design of the study and drafted the manuscript. X-HP, JW, and Y-CT contributed to the analysis and interpretation of data and revised the manuscript. X-BH participated in clinical treatment and literature search. ZW contributed to participated in paper design, paper modification and use of pathological images. All authors read and approved the final manuscript.

## Funding

This study was supported by grants from the National Natural Science Foundation of China (81672902) And the Hospital Research Fund (General Project) from The Second Affiliated Hospital of Army Medical University (no. 2016YLC18 and no. 2019XLC2006).

## Conflict of Interest

The authors declare that the research was conducted in the absence of any commercial or financial relationships that could be construed as a potential conflict of interest.

## Publisher’s Note

All claims expressed in this article are solely those of the authors and do not necessarily represent those of their affiliated organizations, or those of the publisher, the editors and the reviewers. Any product that may be evaluated in this article, or claim that may be made by its manufacturer, is not guaranteed or endorsed by the publisher.
